# Partitioning Social and Spatial Drivers of Infection Risk

**DOI:** 10.1002/ece3.72367

**Published:** 2025-11-02

**Authors:** L. Kirkpatrick, J. Mariën, C. Sabuni, B. Mwamundela, H. Leirs

**Affiliations:** ^1^ School of Environmental and Natural Sciences Bangor University Bangor UK; ^2^ EVECO Universieit Antwerpen Antwerp Belgium; ^3^ Virus Ecology Unit, Department of Biomedical Sciences Institute of Tropical Medicine Antwerp Belgium; ^4^ Pest Management Centre Sokoine University of Agriculture Morogoro Tanzania

**Keywords:** biologging, disease transmission, INLA, proximity logging, social‐spatial modelling, maambukizi ya magonjwa, panya shamba, kiweka kumbukumbu za ukaribu, muundo wa kijamii—anga, wanyama

## Abstract

The rate at which individuals contact each other is central to the transmission of diseases through populations. Most simple models assume homogenous mixing, with all individuals equally likely to contact each other within a population, where contact can either scale with density (the more individuals the more likely contact will occur) or scale independently of density (where individuals maintain contacts regardless of density). However, there is growing evidence that contact rates are non‐homogenous, and both spatial and social structuring are likely to play an important role in producing and maintaining heterogeneous contact behaviour. Additionally, assuming homogenous mixing is a deliberate simplification, yet it can undermine a model's predictive power when spatial or social structure is important, as is often the case for many wildlife diseases. Here, we investigated the relationship between measures of social and spatial behaviour in a non‐territorial rodent species ( *Mastomys natalensis* ) which exhibits significant seasonal fluctuations in density and exposure to a mammarenavirus, Morogoro virus (MORV), using an extensive and unique capture‐mark‐recapture dataset. We followed this up with a study to investigate the extent to which spatial overlap may correlate meaningfully with contact rates using miniaturised proximity loggers developed in house. Exposure to MORV was strongly associated with the proportion of home range overlap with other exposed individuals, and negatively associated with the proportion of home range overlap with conspecifics regardless of exposure status. Including spatial autocorrelation suggested that consistent spatial structuring across the study area also played an important role in determining exposure to MORV. Finally, our proximity logger experiment demonstrated that home range overlap may overestimate direct contact behaviour in *M. natalensis* as individuals showed a high degree of spatial overlap, but most contacts were associated with a small number of individuals.

## Graphical abstract

Contact rates drive disease spread, yet are often oversimplified as uniform across individuals. Using data on *Mastomys natalensis* , we show that exposure to Morogoro virus is linked to spatial overlap with infected conspecifics, but not overall overlap, and that direct contact occurs with only a few individuals despite broad home range overlap—highlighting the importance of spatial and social structure in predicting transmission.

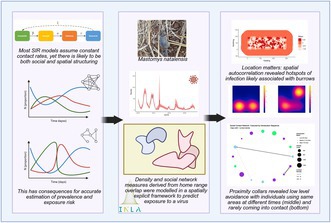

## MUHTASARI

Kiwango cha wanyama kuwasiliana ni muhimu kwa maambukizi ya magonjwa kupitia idadi ya wanyama. Miundo mingi rahisi hudhani mchanganyiko wa wanyama wa aina moja, huku wote wakiwa na uwezekano sawa wa kuwasiliana, ambapo kuwasiliana kunaweza kupimwa kutokana na msongamano (kadiri ya wanyama wengi zaidi uwezekano wa kuwasiliana hutokea) au ukubwa bila msongamano (ambapo wanyama hudumisha mawasiliano bila kujali msongamano). Hata hivyo, kuna ushahidi unaoongezeka kwamba viwango vya mawasiliano havilingani, na muundo wa anga na kijamii una uwezekano wa kuwa na jukumu muhimu katika kuzalisha na kudumisha tabia tofauti za mawasiliano. Zaidi ya hayo, kudhani kuwa mchanganyiko wa aina moja ni kurahisisha kimakusudi, lakini inaweza kudhoofisha uwezo wa utabiri wa mifano wakati muundo wa anga au kijamii ni muhimu kwa magonjwa mengi ya wanyamapori. Hapa, tulichunguza uhusiano kati ya hatua za tabia ya kijamii na anga katika jamii ya panya wasio wa eneo (panya shamba) ambayo huonyesha mabadiliko makubwa ya msimu kwa uwingi na kukabiliwa na virusi vya mammarena, virusi vya Morogoro (MORV), kwa kutumia mbinu kubwa na ya kipekee ya kunasa—kuweka alama—kunasa tena. Tulifuata hili na utafiti ili kuchunguza ni kwa kiasi gani mwingiliano wa anga unaweza kuwiana vyema na viwango vya mawasiliano kwa kutumia teknolojia ya vinasa mawasiliano (kiweka kumbukumbu za karibu). Kukaribiana kwa MORV kulihusishwa sana na uwiano wa masafa ya nyumbani na wanyama wengine walio wazi, na kuhusishwa vibaya na uwiano wa masafa ya nyumbani na vipimo maalum bila kujali hali ya kukaribia aliyeambukizwa. Ikiwa ni pamoja na urekebishaji wa anga ulipendekeza kuwa muundo thabiti wa anga katika eneo lote la utafiti pia ulichukua jukumu muhimu katika kubainisha kukaribiana kwa MORV. Hatimaye, jaribio letu la kiweka kumbukumbu za ukaribu lilionyesha kuwa mwingiliano wa masafa ya nyumba unaweza kukadiria kupita kiasi tabia ya mawasiliano ya moja kwa moja katika panya shamba kwani wanyama wengine walionyesha kiwango cha juu cha mwingiliano wa anga, lakini mawasiliano mengi yalihusishwa na idadi ndogo ya wanyama.

## 1 Introduction

A central focus in the study of disease transmission dynamics is around understanding the influence of host behaviour on pathogen spread. For directly transmitted pathogens, the transmission coefficient, often denoted as *β*, is used to represent the interplay of two primary processes (McCallum et al. 2017). Beta can be decomposed into the contact rate—the frequency at which susceptible individuals come into contact with infectious individuals—and the probability of transmission given that contact has occurred, with the result that β is dependent on both behaviour (contact occurring) and physiology (transmission occurring given contact; McCallum et al. 2017; Vanderwaal and Ezenwa 2016). Consequently, both the nature and duration of contact influence transmission probability (Vanderwaal and Ezenwa 2016), with their relative importance depending on the pathogen's transmission pathway—for example, whether spread occurs via direct physical contact, prolonged proximity, or brief environmental exposure.

At the population level, disease transmission models typically capture the progression of individuals through susceptible, infectious, and recovered states (SIR). Most SIR models assume homogeneous contact rates between individuals, where contact is thought to occur either independently of density (frequency‐dependent) or to scale linearly with density (density‐dependent; McCallum et al. 2017). This assumption has potentially serious implications for understanding disease transmission in populations (Hopkins et al. 2020; White et al. 2017). However, there is growing evidence that representing contact rates solely as a linear function of density may not accurately capture real‐world transmission processes (White et al. 2017; Hopkins et al. 2020; Albery et al. 2025). For instance, contact behaviour may exhibit a sigmoidal relationship with density (Borremans, Reijniers, Hens, and Leirs 2017), with implications for pathogen transmission (Borremans, Reijniers, Hens, and Leirs 2017; Hu et al. 2013). In fact, the more density fluctuates, the more crucial it becomes to choose an appropriate transmission coefficient (Borremans, Reijniers, Hens, and Leirs 2017). If transmission is strictly density‐dependent, pathogen prevalence would be expected to decline or even vanish in populations where densities fall below a critical threshold (Borremans, Reijniers, Hens, and Leirs 2017). However, if individuals adjust their behaviour at low densities to maintain sufficient social contacts, pathogens may persist (Hopkins et al. 2020). Understanding whether such behavioural adjustments occur is essential for the effectiveness of public or wildlife health interventions, such as culling wildlife reservoirs (Hopkins et al. 2020; Vicente et al. 2007; Mariën et al. 2019).

In addition, the spatial scale at which interactions occur may influence interpretations of the contact function, with density‐dependent relationships at the local scale and a more frequency‐dependent relationship at the population scale (McCallum et al. 2017; Lunn et al. 2022). Many models rely on assumptions of homogeneous and random mixing (McCallum et al. 2017), which is often unrealistic; animals typically interact more frequently with those in close spatial proximity than with distant conspecifics (White et al. 2017). However, long‐distance interactions may still play significant roles in pathogen transmission (Drewe 2010) and are often driven by non‐random social preferences (Vanderwaal and Ezenwa 2016; Albery et al. 2025) or intrinsic individual differences. Additionally, animal ranging patterns and contact behaviours can vary over time in response to resource availability (Springer et al. 2017). Incorporating such heterogeneity is essential for developing more realistic and accurate models of disease transmission.

Social network analyses, which explicitly examine heterogeneities in contact behaviour (the frequency, duration, and type of interactions between individuals; Sah et al. 2018), are increasingly providing insights into how such behavioural variation influences disease transmission (Perkins et al. 2008). These networks represent individuals as ‘nodes’ and the interactions between individuals as ‘edges’ (Sah et al. 2018; Croft 2016; James et al. 2009); in the context of directly transmitted diseases, edges represent potential contacts between individuals. Generally, individuals with a higher number of social connections (high degree) or that link different subgroups (high centrality) are likely to play a more significant role in disease transmission (Sah et al. 2018). Social networks not only characterise contact behaviour but can also be weighted by the nature and duration of contacts, providing insights into the types of contact necessary for transmission (Farine and Whitehead 2015). Including this information is crucial, as pathogen risk is frequently cited as a major cost of group living (Webber and Vander Wal 2020), with individuals in larger social groups facing higher parasite or pathogen burdens than those in smaller groups (Webber and Vander Wal 2020). For instance, in giraffes, individuals with more social ties to other groups exhibit higher helminth infection levels (Vanderwaal and Ezenwa 2016), and in meerkats, *Mycobacterium bovis* transmission is associated with increased in‐group interactions and intergroup roving behaviour in males (Drewe 2010).

While social networks offer valuable insights into how individual heterogeneity in behaviour influences disease transmission, they often either overlook spatial effects or treat them solely as confounding factors to be controlled for, rather than integrating them as an explicit component of analysis (He et al. 2019). This is despite the inherently spatial nature of social contacts; individuals can only interact with those in close proximity (He et al. 2019; Farine and Whitehead 2015), and such interactions may be driven more by environmental resources than by social preferences (He et al. 2019). For example, individuals may gather preferentially at key resources regardless of the identity of nearby conspecifics (He et al. 2019). Alternatively, they may selectively seek out other individuals based on relatedness or mate‐seeking behaviour (Stockmaier et al. 2021; Sosa, Jacoby, et al. 2021). Habitat structure or fragmentation may also limit contacts between groups due to spatial constraints rather than adaptive social processes. Misattributing these limitations to social preferences can lead to erroneous assumptions about adaptive behaviours, such as concluding that distinct social communities are formed to reduce disease spread rather than as a product of habitat structure (He et al. 2019). Integrating spatial processes into transmission models can improve strategies for surveillance, containment, and mitigation (Pepin et al. 2021; Pandey et al. 2024). In non‐vector‐borne diseases, both social and spatial processes are likely to influence contact heterogeneity (Pepin et al. 2021; Pandey et al. 2024), yet our understanding of spatial factors remains relatively limited (Pepin et al. 2021).

In addition to assuming homogenous mixing, most disease transmission models also assume all transmission events are observed (Pepin et al. 2021), which is often not the case. The extent to which coarse‐scale capture‐mark‐recapture data accurately represents contact data at biologically relevant scales is frequently unclear (Gilbertson et al. 2021). For small animals, where accurate contact data collection is challenging, inferring contact based on spatial overlap may under‐ or overestimate contacts, particularly if sampling intervals do not align with species‐specific behaviours such as mating (Gilbertson et al. 2021). Using technological approaches such as proximity loggers can add context to long‐term capture‐mark‐recapture data by offering short‐term insights into animal associations. Therefore, we included an experiment to investigate wild animal contact behaviour over a short duration (2 weeks) to better understand contact patterns at finer spatiotemporal scales. Biologgers that record proximity have been used in studies to identify spatiotemporal overlaps (e.g., Racoons: Hirsch et al. 2013; badgers: Drewe et al. 2013) but have been restricted in use due to their large size and short battery life. We used newly developed miniaturised proximity loggers capable of recording high‐resolution contacts (e.g., every 2 min) for up to 2 weeks (Kirkpatrick et al. 2021; Huels et al. 2025) to assess contact rates alongside coarse movement data from loggers placed in the environment. Findings from this data were used to complement spatiotemporally explicit modelling using a long‐term capture‐mark‐recapture dataset to ask the following question: Can we disentangle the role of social vs. spatial factors in driving patterns of exposure to a virus?

## 2 Methods

### 2.1 Study Site and Species

The multimammate mouse, *Mastomys natalensis* , a widespread rodent throughout sub‐Saharan Africa, is recognised as a significant agricultural pest and a reservoir host for several zoonotic pathogens, including *Yersinia pestis* (causative agent of plague), Lassa mammarenavirus, and Leptospira (Leirs et al. 2023). In Tanzania, where field data for this study were collected, *M. natalensis* populations undergo notable seasonal fluctuations, with densities ranging from approximately 10 individuals per hectare during the breeding season to as high as 150 individuals per hectare outside the breeding season. Population dynamics have been closely monitored using Pollock's robust design (Pollock 1982) in open capture‐mark‐recapture since 1994 (Leirs et al. 2023), and the presence of a non‐pathogenic mammarenavirus, Morogoro virus (MORV), has been tracked in this population since 2010 by screening for MORV antibodies (Borremans, Vossen, et al. 2015). MORV is transmitted horizontally and vertically (Hoffmann et al. 2021; Borremans, Vossen, et al. 2015), both through direct contact (Mariën et al. 2020) and environmental contamination (faeces, urine; Hoffmann et al. 2021; Borremans, Vossen, et al. 2015). Adult acquired infections are typically acute, with viral shedding lasting around 18–40 days (Borremans, Vossen, et al. 2015). In contrast, vertical transmission from mother to offspring results in chronic infection and lifelong shedding, contributing to the persistence of MORV through periods of low density (Borremans, Vossen, et al. 2015; Hoffmann et al. 2021; Mariën et al. 2020). Antibody responses are generally considered to be long‐lasting, with only limited and infrequent evidence of seroreversion (Borremans, Vossen, et al. 2015).

### 2.2 Field Data Collection

A subset of the long‐term capture–mark–recapture (CMR) dataset was used for this study, restricted to the period between January 2010 and April 2017 to coincide with the availability of seroprevalence data. The study site, MOSA, is situated just outside Sokoine University of Agriculture, Morogoro, and consists of 300 single capture Sherman traps deployed in a 300 × 100 m grid with a 10 m gap between each trap. Traps are baited with a mixture of peanut butter and cornflour and are deployed for three consecutive nights each month. Captured individuals are weighed, assessed for sex and reproductive status, and uniquely marked with a toe‐clipping code at first capture (Borremans, Sluydts, et al. 2015). A blood sample was collected retro‐orbitally using a 30 μL capillary tube and stored on Whatman filter paper for subsequent MORV antibody screening (Mariën et al. 2020). Filter papers were initially stored at −4°C and later moved to −20°C for long‐term storage.

### 2.3 Density Estimation

Population density was estimated using spatially explicit capture‐mark‐recapture (SECR) models implemented in the R package ‘secr’ (Efford 2023). SECR models integrate a spatial point process with an observation process to jointly estimate population density without bias (Efford 2023). Initial sigma (*σ*), which describes the spatial scale of animal movements and determines how detection probability declines with distance from the activity centre, was estimated using the RPSV function, with a buffer set at four times the initial sigma estimate. The detector type was set to ‘multi’ because no likelihood function currently exists for single‐use traps in the secr package (Efford 2023). Furthermore, simulation studies have demonstrated that multi‐catch trap models produce density estimates comparable to those from single‐catch traps. Additionally, occasional captures of multiple individuals in the same trap in our study support this approach. To capture temporal variation in population density at a monthly scale, session‐specific SECR models were fitted separately for each monthly trapping session within a robust design framework. This approach allowed estimation of density changes over time while accounting for demographic processes such as birth, death, and temporary emigration between sessions. A half‐normal detection function was used, reflecting the rarity of long‐distance detections of *Mastomys natalensis* in this system (Leirs et al. 1996). A behavioural response parameter was included to account for changes in capture probability following repeated trap exposure.

### 2.4 Social Network Measures

Contact networks were constructed using home range overlap, which requires estimating the spatial behaviour of individuals. Only individuals trapped in two or more trapping sessions were included, as home range size and location cannot be reliably estimated from a single detection. We defined home range as 100% minimum convex polygons bounded by the locations at which an individual was trapped following Borremans, Reijniers, Hughes, et al. (2017), assuming that it is a proxy for the space used by an animal during its daily activities such as foraging, mating, or litter care (Borremans, Reijniers, Hughes, et al. 2017). MCPs were estimated with a 5 m boundary strip as described in Borremans, Reijniers, Hughes, et al. (2017). Due to declining recapture rates and population density over the study period, we opted for 100% MCPs rather than kernel utilisation distributions, as the lower number of relocations per individual limited the reliability of kernel‐based methods, which require more repeated spatial data to accurately estimate core areas of use. Individuals were assumed to have a ‘contact’ when both were trapped in the same 2‐month window and their overall home ranges (calculated over the full duration of the study period) overlapped.

The following network measures were calculated for each individual and included in statistical analyses: *Degree*, the total number of contacts as determined by home ranges intersecting within a 2‐month window, and *strength*, the total number of contacts within a 2‐month window weighted by the extent of home range overlap. Degree captures the total number of potential contacts within a network, while strength reflects the cumulative extent of home range overlap with those contacts, which may better represent the spatial opportunity for disease transmission. For example, some individuals may associate more frequently with a small number of individuals within a larger cohort of social contacts (Sosa, Jacoby, et al. 2021). *Betweenness centrality* was calculated as the number of times an individual appeared on the shortest path calculated between every combination of two nodes (Sosa, Sueur, and Puga‐Gonzalez 2021) and captures how position in the social network may influence disease flow (Sosa, Sueur, and Puga‐Gonzalez 2021). Individuals with a high betweenness score may link otherwise separate subgroups and play a key role in disease persistence across a network (Silk et al. 2017). *Eigenvector centrality* was calculated as the first non‐negative eigenvector value obtained by the linear transformation of the adjacency matrix and takes into account not only how connected the focal individual is but also how connected the individuals the focal individual is connected to are (Sosa, Sueur, and Puga‐Gonzalez 2021; Silk et al. 2017). Finally, *positive strength* was calculated as the weighted proportion of home range overlap with a seropositive individual within a 2‐month window (Table 1).

**TABLE 1:** Description of the fixed effects included in the spatially explicit GLMMs.

Measure	Description	Retained in final model?	Mean and range
Degree	Number of nodes a node is connected to as determined by home range overlap	No	9.4 (0–25)
**Strength**	Number of nodes a node is connected to as determined by home range overlap, weighted by the proportion of home range overlap	Yes	4.7 (0–12.3)
**Positive strength**	Number of positive nodes a node is connected to as determined by home range overlap weighted by the proportion of home range overlap	Yes	1.3 (0–9)
Betweenness	Number of times an individual appeared on the shortest path calculated between every combination of two nodes	No	182.6 (0–1353)
Eigenvector centrality	How connected an individual, and an individuals connections are within the network	No	0.16 (0–8.87)
Reach	Proportion of nodes an individual can reach by following edge paths. A fully connected graph would have a reach of 1.	No	
Minimum length	Minimum distance to an infected individual as measured between centroids	No	
**Age**	Maximum number of months an individual is detected in the dataset at each time point	Yes	2.39 (1–7)
**Obs**	Number of times an individual is captured	Yes	2.08 (1–7)
**Density**	Estimated number of *M. natalensis* per hectare	Yes	31.6 (1–73)
Lagged density	Density at *t* − 1	No	
Sex	Sex of the animal	No	

*Note:* Measures retained in the final model are highlighted in bold.

### 2.5 Bayesian Spatiotemporal Model

The relationship between exposure to MORV (response variable), the various social network measures, and individual characteristics (fixed variables) was modelled using a logistic regression model within a Bayesian framework using Integrated Nested Laplace Approximation (INLA). This was fitted using the linear modelling package R‐INLA (Rue et al. 2009) in R version 4.1.2 (R Core Team 2021). Exposure to MORV, as determined by the presence of antibodies, was used as the response variable (unexposed, exposed), and fixed explanatory variables included density (both current and lagged by 1 month, mice per hectare), age (estimated, in months), sex, and mass (g) of the individual, as well as measures derived from contact networks (Table 1). Individual ID was included as a random effect in all models to account for repeated captures, and a seasonal autocorrelative temporal effect was included for temporal models. For the spatial and spatiotemporal models, INLA allows for the incorporation of a spatially distributed random effect using a stochastic partial differential equation approach (Bakka et al. 2018). This approximates a continuous random field by using a mesh of connected, discrete locations (Rue et al. 2009). The mesh can then be used to identify ‘hotspots’ by plotting the response variable in 2D (e.g., Figure 3), and the distance over which autocorrelation can be detected can be extracted via the kappa/range parameters. Spatiotemporal INLA models were constructed by allowing spatial fields to vary according to a temporal autocorrelative model; we compared an autoregressive 1 seasonal spatiotemporal effect, a random walk seasonal spatiotemporal effect, an exchangeable spatiotemporal effect, and a fixed spatiotemporal effect (see Table 2 for full model specifications and Bakka et al. 2018 for details). Model sets comprised the following: fixed effects only, fixed + temporal effects, fixed + spatial effects, fixed + spatiotemporal effects, and no fixed effects (see Table 2 for full details of the different models). Models were compared using the Watanabe Akaike Information Criteria (WAIC, Gelman et al. 2014); a reduction of 2 or more in WAIC was used to select the best fitting model. All R code and a subset of the data are included in the Supporting Information.

**TABLE 2:** Comparison of WAIC, change in WAIC, and effective number of parameters for the full model set.

Model	WAIC	ΔWAIC	pEff_Waic
Fixed effects only			
All fixed effects	951.28	3.39	59.5
Significant effects only*	955.53	7.64	32.23
Inclusion of temporal autocorrelation with fixed effects			
Fixed + Year	965.95	18.06	12.52
Fixed + Season	959.08	11.19	29.65
Inclusion of spatial and temporal autocorrelation with fixed effects			
**Fixed + Spatial**	**947.89**	**0**	**87.86**
**Fixed + Spatial + Seasonal**	**949.72**	**1.83**	**89.84**
Inclusion of spatiotemporal autocorrelation with fixed effects			
Fixed + Spatiotemporal IID	966.3	18.41	27.29
Fixed + Spatiotemporal EXC	951.68	3.79	80.17
Fixed + Spatiotemporal RW	2061.6	1113.71	200.81
Fixed + Spatiotemporal AR1	951.49	3.6	83.69
Fixed effects excluded			
Spatial + Seasonal	1569.26	621.37	256.86
Spatial only	1590.96	643.07	241.16
Seasonal only	1703.38	755.49	406.04

*Note:* Bold font indicates the preferred models (best fit according to DeltaAIC).

* Significant only fixed effects refers to those highlighted in bold in Table 1.

### 2.6 Investigating Direct Contact Behaviour

Direct contact behaviour was investigated using newly developed miniature proximity loggers (IoSA BV, Belgium; Kirkpatrick et al. 2021; Berkvens et al. 2019; Huels et al. 2025). The proximity logger system uses Bluetooth Low Energy (BLE) technology, where each logger acts as both a transmitter and receiver. When one logger comes within range of another, the receiver records the timestamp, received signal strength (RSSI), and identity of the contacting logger, enabling detailed measurement of contact events. Loggers were fitted to rodents as a collar constructed from a cable tie coated in heat shrink. Loggers were coated in epoxy resin to protect against damage from rodents. Collars weighed between 2.1 and 2.7 g and did not exceed 8% of the rodents' body weight. From previous trials with *M. natalensis* , and calibrating within the complex habitat structure in our enclosures, we classified contacts recorded with a received signal strength greater than or equal to −80 dB as a potential contact, as this would suggest that the two mice came within 1 m of each other within the 120 s sampling interval. Eleven stationary loggers were placed throughout the enclosure in a regular grid, mounted on 2 m poles. Stationary loggers can detect mobile loggers up to 15 m when above ground and were used to confirm mobile logger functionality during the study period. Additional analyses comparing stationary logger detection and contact behaviour are part of ongoing work and beyond the scope of this study.

The proximity loggers work by alternately scanning for other loggers in the vicinity and advertising their own ID. The rate at which loggers scan and advertise is user determined; in our case, we set mobile loggers to scan every 2 min with the advertising interval of 430 ms. This advertising interval was automatically calculated based on optimising the battery lifespan. Loggers were set to hibernate between 10 am and 4 pm as *Mastomys natalensis* is nocturnal. Stationary loggers, which have a larger battery, were set to scan for mobile loggers every 10 s for 24 h a day. We considered any log recorded on the stationary loggers as a contact, regardless of received signal strength.

*Mastomys natalensis* used in the collaring study were trapped originally from agricultural fields in the vicinity of the enclosures using live Sherman traps baited with a mixture of peanut butter and flour placed in a regular grid. Traps were set after 15:00 in the afternoon and collected at 7:00 the following morning. Mice were then released into the enclosure and left for 2 weeks, after which they were trapped using the same methods as before. Trapped mice were taken to the laboratory where they were weighed, sex, and reproductive status recorded, and collars were attached. Only mice weighing above 40 g were selected for this experiment. Mice were held for 24 h following collar attachment to ensure that collars did not cause any problems and that they couldn't be removed. Mice were then released the following afternoon just before sunset into an enclosure at the field site at Sokoine University of Agriculture, Morogoro. The enclosure measures 35 m × 35 m and is constructed out of metal sheeting to prevent mice from escaping or entering. The habitat inside is a mixture of shrubs, bushes, and grass. Mice were released at the locations at which they were trapped at three 5‐day intervals, mimicking the increase in population density in the dry period. A 5‐day gap between releases was chosen to stay within the predicted battery life of the loggers. Initially, six mice were released at the first time point, then another 12 at each later time point, resulting in a total population of 30 mice. The sex ratio of the mice reflected that of the wild populations from which mice were trapped. The loggers can be remotely downloaded onto an SD card through the use of a gateway which was placed in the centre of the enclosure; the grid was checked daily by checking and downloading all stationary loggers and by walking a regular route with the gateway to download any mobile loggers. In addition, the enclosures were swept with the mobile phone app to search for any collars that had been removed or mice that had been predated. If mice were discovered, information about their location was recorded. Any mice discovered dead were identified using their unique toe clipping if their collar was not present; any collars discovered removed from mice were also collected and any data downloaded. After 15 days, traps were placed for 1 week to trap back in collared rodents and remove and download any remaining collars. All experiments were carried out following the ethical guidelines of Sokoine University of Agriculture.

The presence and movement of mice across the grid was visualised in R, using heat maps. Heat maps were calculated using the spatstat package (Baddeley and Turner 2005) by creating spatial density plots based on the *X* and *Y* coordinates of the stationary loggers. A social contact network was constructed using igraph (Csardi and Nepusz 2006) and ggraph (Pedersen 2025). Given that mice were in the arena for different lengths of time, and there was not constant detection probability, we opted for a hybrid approach to calculate contact rates. First, we removed any individuals who were either never seen in the grid (potentially escaped/predated or logger failure) or those who were seen for less than 24 h, as we could not account for whether these animals were still present. Next, we estimated the consistency with which an individual was detected by calculating the period in which an animal was present as determined by detections on the stationary loggers, and the proportion of days each individual was detected during this period. For each dyad, we then determined their temporal overlap period as the intersection of the periods in which they were present, and adjusted this by their average detection rate. This allowed us to therefore estimate the number of days both dyad members were present and available for contact, accounting for periods during which individuals may have been present but undetected due to coverage limitations. We then divided the total contacts for each dyad by their adjusted overlap period to give a measure of contact density. In this way, we could distinguish between dyads which had a short but intense interaction compared to dyads with a longer but less intense interaction.

## 3 Results

**FIGURE 1:**
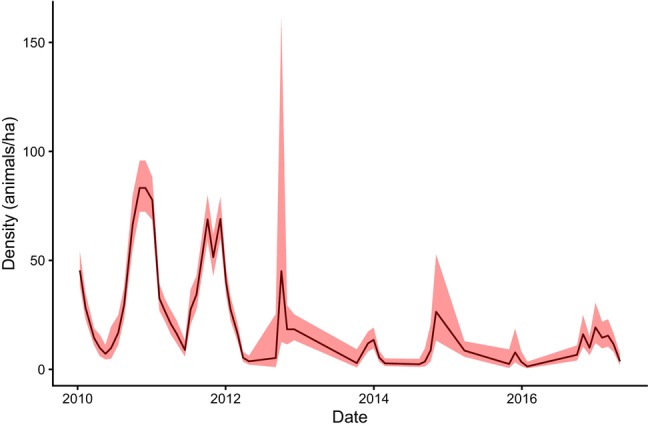
Changes in density as predicted by the spatial capture recapture model from January 2010 to December 2018. Red shading indicates the 95% confidence intervals. Density is measured in individuals per hectare.

The densities of *M. natalensis* fluctuated periodically until 2012, after which a marked and sustained decline in mean population density was observed (Figure 1). Estimated densities ranged from a minimum of 1.35 (0.52–3.52) to a maximum of 83 (72–160) individuals per hectare. Following 2012, population fluctuations diminished substantially, and mean density dropped to levels lower than those recorded before 2012 (pre‐2012 mean: 30 individuals/ha, 95% CI: 25–42; post‐2012 mean: 12 individuals/ha, 95% CI: 7–22).

### 3.1 Spatially Explicit Models

**FIGURE 2:**
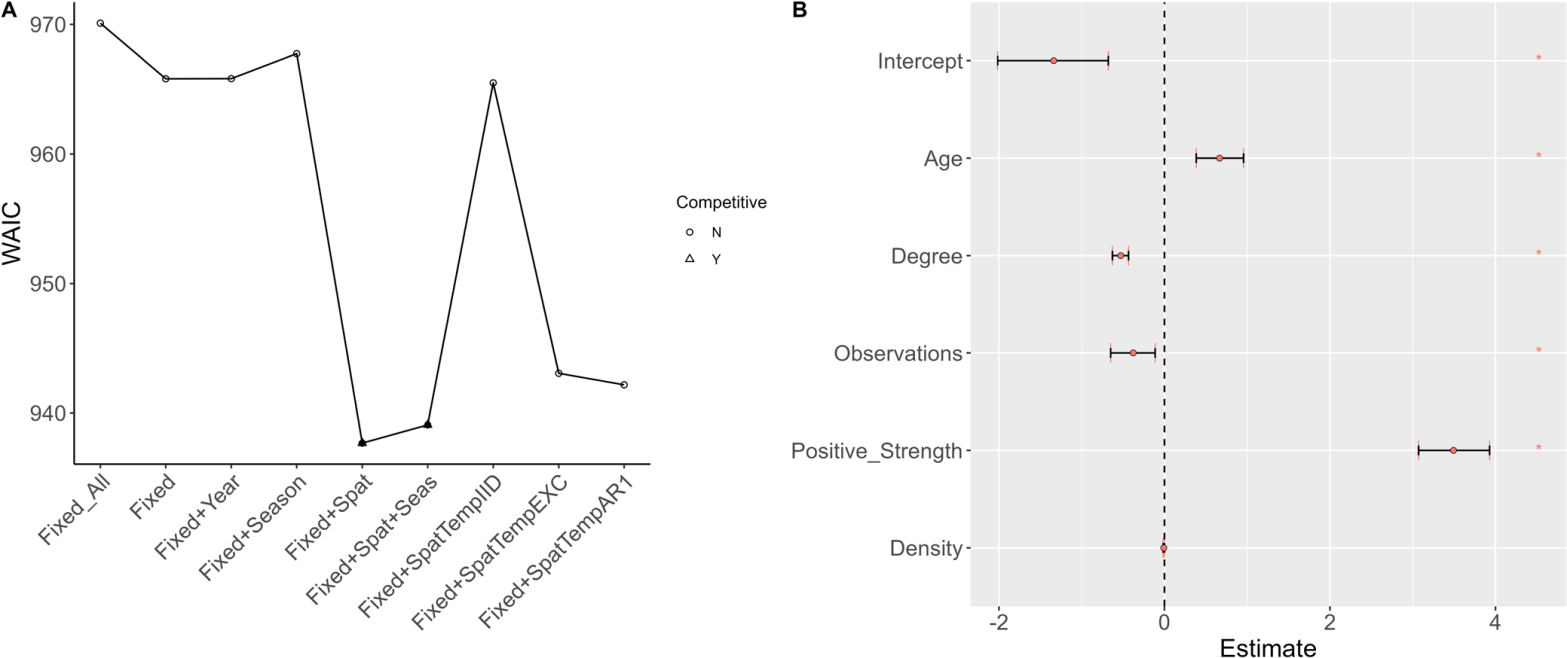
(A) Comparison of the WAIC for all models tested apart from four models excluded due to extreme WAIC values (Table 2, Table S1). The filled triangles indicate the two best fitting models as determined from comparison of WAIC. (B) Estimates and effect sizes from the best fitting model which included the fixed effects (Table 1) and a fixed spatial term. The figure shows the influence of each variable on exposure to MORV. Significance is determined by assessing whether the credible intervals (whiskers) around the mean (points) cross zero (indicated by the dashed vertical line). Points and whiskers below zero (to the left of the dotted line) indicate a negative influence on MORV exposure, while points and whiskers above zero indicate a positive effect of MORV on exposure.

The capture mark recapture dataset contained 3322 unique records, spanning 2010–2017 and 197 unique trapping sessions. Gaps in data collection after 2012 are a consequence of a reduction in *M. natalensis* population size, resulting in fewer individual recaptures and consequently fewer animals available for home range estimation. The best fitting spatially explicit model of MORV exposure included individual fixed effects and a constant spatial field across the entire sampling period (Figure 2A). The probability of being exposed to MORV was strongly positively associated with a large home range overlap with other exposed individuals (positive strength, Figure 2B, Table 3) and negatively associated with home range overlap with all individuals (Degree, Figure 2B, Table 3). Older individuals (as determined by the number of months between the last and the first time an individual was captured) were more likely to have been exposed to MORV (Age, Figure 2B, Table 3), while there was a negative association between the number of times observed and the probability of being exposed to MORV (Obs, Figure 2B, Table 3). Finally, population density was negatively associated with the probability of MORV exposure, although the magnitude of the effect was very small (Density, Table 3).

**TABLE 3:** Estimate mean and credible intervals for the best fitting model showing the fixed effects retained within the model, all of which are significant based on the credible intervals not overlapping zero.

	Mean	SD	0.025 CI	0.975 CI
Intercept	−1.34	0.34	−2.03	−0.69
Age	0.66	0.15	0.38	0.95
Strength	−0.53	0.05	−0.63	−0.43
Times observed	−0.37	0.14	−0.65	−0.11
Strength positive	3.49	0.22	3.07	3.94
Density	−0.01	0	−0.02	−0.002

**FIGURE 3:**
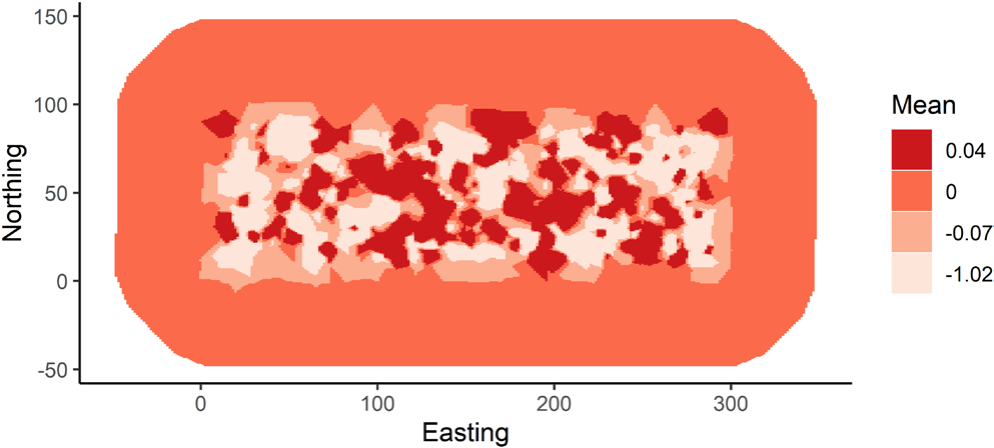
The spatial field of variation showing the hot and cold spots of the probability of exposure to Morogoro virus. The spatial field is predicted from the top fitting model which included a fixed spatial field over the entire dataset, therefore this plot shows the predicted, temporally stable areas of increased (dark red) and decreased (white) risk. The dark spots show ‘hotspots’ of positivity while the lighter areas show ‘cold spots’ where individuals are less likely to be exposed to MORV.

The two best fitting models (ΔWAIC < 2, Figure 2A) both included a spatial field that was constant across the study period. The range of spatial autocorrelation estimated from the model was small (< 10 m) and suggests that spatial structuring is operating over relatively short distances (Figure 3). A constant spatial field was selected over a seasonally varying spatial field, suggesting that the spatial distribution of hotspots of exposure to MORV was spatially consistent over the entire sampling period and did not vary temporally. Although the second‐best fitting model included a seasonal temporal autocorrelation structure, the mean and confidence intervals for this model were extremely similar to those of the best fitting model; hence, the less complex model is preferred.

### 3.2 Direct Contact Behaviour

**FIGURE 4:**
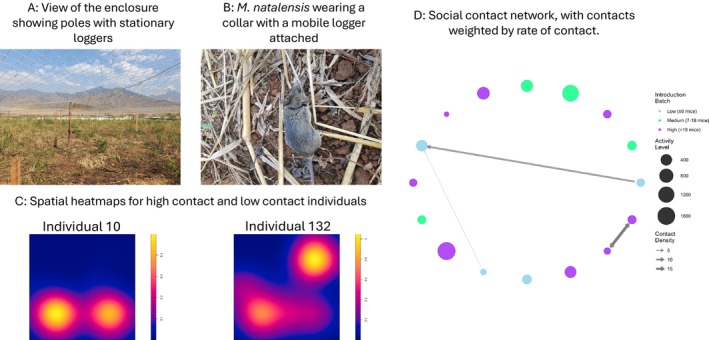
Description of the logger trial showing the enclosure in which the experiment was carried out (A) and a Mastomys natalensis wearing a collar with the logger attached (B). (C) shows spatial maps plotted for two individuals, one of which had a large number of contacts (individual 10) and the other no contacts (Individual 132). Colour intensity indicates the number of logs recorded by stationary loggers at those locations. Spatial scale for both plots is the entire enclosure (35 m × 35 m). (D) shows social networks for the mice. Nodes are coloured by the time of introduction corresponding to the density of mice at each time point; blue nodes represent low density of mice, green represent medium density of mice while purple represents high density of mice. The size of the node indicates the number of contacts recorded by the stationary loggers, giving an indication of activity across the grid. Edge width represents contact density (contacts per estimated day of co‐presence), calculated using a hybrid approach that adjusts for detection probability.

On *M. natalensis* , the proximity loggers can detect another logger up to around 2 m depending on the complexity of the habitat structure, while stationary loggers can detect up to a distance of 10 m depending on habitat structure. Stationary loggers cannot detect mobile loggers that are underground, and their range is reduced if mobile loggers are in dense vegetation. Therefore, despite having a regular grid in the enclosure, we were not able to accurately map rodent movements at all times. Our animal‐mounted loggers recorded a total of 2348 contacts, while our stationary loggers distributed in the environment recorded a total of 7336 contacts. Stationary loggers picked up 18 of the 30 mice that were tagged and released, but contacts were recorded between far fewer individuals (5 of 30 individuals) and overall remained low. When contacts were weighted by the number of proximity events detected between individuals and the duration of contact logging to create a ‘rate of contact’ metric, contacts between individuals were only recorded during low and high density periods (Figure 4). Stationary loggers picked up more individuals than were detected on mobile loggers (Figure S1), and spatial heatmaps (e.g., Figure 4C) showed that movement between individuals varied. Some individuals were only picked up in one area of the enclosure and appeared to only move within the vicinity of that stationary logger; others were picked up at multiple stationary loggers suggesting longer range movements across the grid (Figure S1). Variation in both contact behaviour and spatial movements was seen; for example, a high contact individual, ID10, contacted two other individuals, including during the day by sharing a burrow with another individual, but occupied a smaller area of the enclosure than ID132, which did not contact other individuals but ranged further over the study area (Figure 4). The Supporting Information contains additional details about the logger study and the contact detections.

## 4 Discussion

Both spatial and social behaviours will drive movement across the landscape, which generates patterns in social associations (Webber et al. 2023; Pandey et al. 2024; Pepin et al. 2021). Given that these behaviours often arise in tandem, tests of hypotheses that explicitly investigate either social or spatial processes while ignoring or controlling for the other may misallocate behaviours driven by one process to the other (Webber et al. 2023; Albery et al. 2020). In this study, we demonstrate that both social interactions and spatial autocorrelations provide valuable insights into the possible drivers of disease transmission. In addition, we demonstrate how miniaturised loggers can give insights into animal movement and behaviour at a high spatial and temporal resolution.

We found very limited evidence that exposure to MORV is associated with population density, despite this being found in other studies using the same study system (Mariën et al. 2020). While the impact of density was small, it was significant and negative, most likely reflecting the influx of non‐exposed juveniles into the population, the so‐called juvenile dilution effect (Mariën et al. 2020). This could be because including spatial variation modelled through both home range overlap and the inclusion of a spatial field in R‐INLA captures variation related to host movement, behaviour, and local environmental heterogeneity, which can absorb some of the effects otherwise attributed to density fluctuations, particularly if density influences movement behaviour (Borremans et al. 2014; Albery et al. 2020; Vander Wal et al. 2014). Indeed, our logger experiment showed that despite density increases that lead to a high‐density population in our enclosure experiment, most individuals practiced fine‐scale avoidance and rarely came within 1 m of each other despite moving within the same area at the same time (Figure 4D, Figures S1 and S2). Contact rates followed a Pareto distribution with < 20% of individuals responsible for more than 80% of contacts, suggesting that intraspecific variation in contact rates exists for *M. natalensis* . Given that social behaviour may confer benefits in terms of access to resources or increased mating opportunities while also incurring costs of increased disease transmission risk or antagonistic interactions with conspecifics over diminishing resources (Vanderwaal and Ezenwa 2016; Ashby and Farine 2022), it is likely that some individuals will adjust their movement behaviour to continue to avoid conspecifics during periods of high density. Such saturating relationships between density and contact rates appear common across multiple study systems (Albery et al. 2025) and may reflect ecological factors such as increased competition for resources or decreased fecundity during periods of high density (Albery et al. 2025).

The likelihood of being exposed to MORV was most strongly associated with the proportion of an individual's home range that overlapped with that of other exposed individuals, and negatively associated with the degree of home range overlap with individuals regardless of exposure history to MORV. Given that MORV transmission likely has important direct and indirect components, with viral RNA shed in saliva, urine, and faeces (Mariën et al. 2020; Hoffmann et al. 2021), a large home range overlap with other positive individuals could increase transmission risk due to a susceptible individual either encountering an infectious individual or contamination in the environment during movements within the home range. Periods during which home range overlap with other individuals was largest regardless of the exposure status of individuals coincided with periods of recruitment and density increases when the majority of individuals are unexposed juveniles; hence, the risk of contacting an infectious individual would be lower.

We found that a constant spatial field greatly improved model fit (ΔWAIC > 2) compared to a temporally fluctuating spatial field or no spatial field. This suggests that the spatial locations of exposure risk remained consistent over the entire study period and did not fluctuate seasonally as might be expected if environmental drivers such as the presence of maize cropping on certain parts of the study area are driving increased contact rates and exposure risk. This could be explained by two potential mechanisms. Firstly, the spatial structuring could reflect the distribution of burrows in the environment. *Mastomys natalensis* rarely digs its own burrows but often uses other burrows or ground features that have been created by other species. Therefore, these hotspots of exposure may be where certain burrows are in continuous use, and therefore act as key contact points. Alternatively, individuals which are infected as neonates can become chronically infected and will shed MORV throughout their lifespan (Hoffmann et al. 2021; Mariën et al. 2020). Given that *M. natalensis* disperse relatively short distances (Hooft et al. 2008), it is possible that the spatial structuring is due to the presence of chronically infected individuals that persist in their natal home ranges and continue to transmit MORV to susceptible individuals.

It is often assumed that home range overlaps derived from capture‐mark‐recapture studies may more accurately reflect social networks when density is high rather than low (Perkins et al. 2009). However, if avoidance of conspecifics is occurring at the microhabitat scale, as we see happening in our enclosure study, home range overlap may overestimate contact probability during periods of high density, with consequences for the understanding of disease transmission. This will be more significant for direct rather than indirectly transmitted pathogens, where spatial overlap in movements may better reflect contact with a contaminated environment (e.g., through urine or faeces; Pandey et al. 2024). Previous studies have demonstrated that home range overlap can correlate well with contact rates in certain species, but the extent to which this is true can be affected by other factors such as seasonality (e.g., raccoons are more gregarious in winter than in summer; therefore, winter home range overlaps are more representative of contact rates than summer home range overlaps; Robert et al. 2012) or dyad composition (McIlraith et al. 2025).

The choice of index representing home range overlap is also important; measures such as the utilisation distribution overlap index (a probability distribution defining the animals' use of space; Fieberg and Kochanny 2005) more accurately correlated with contact rates than other measures (Robert et al. 2012), particularly when the 95% isopleth was considered. Therefore, exploring different indices of home range overlap that take into account the proportion of time spent in different home range areas may give a better representation of contact rates than approaches such as home range overlap proportions (Fieberg and Kochanny 2005). While other home range overlap metrics, such as utilisation distribution‐based measures, can more accurately reflect potential contact rates by focusing on areas of concentrated activity and thus avoiding overestimation inherent in 100% minimum convex polygons (MCPs), they require higher‐resolution spatial data and frequent recaptures. Consequently, these methods may not be suitable in studies with low recapture rates or sparse spatial data, where simpler approaches like 100% MCPs provide a pragmatic, albeit conservative, estimate of overlap. Furthermore, the extent to which large‐scale overlaps in movement (as determined by home range overlaps) and fine‐scale interactions (e.g., close contact behaviour) may correlate can be biased by other factors such as relatedness (McIlraith et al. 2025), age‐related differences in movement (Mariën et al. 2020), or social behaviour (e.g., changes in behaviour based on sexual maturity, personality (Vanden Broecke et al. 2017), or sickness; Stockmaier et al. 2021). The correlation between measures of home range overlap and contact rates may not be consistent both within a species or within an individual's lifetime, with consequences for the impacts of contact heterogeneity on processes such as disease spread (Robert et al. 2012; Vander Wal et al. 2014). Indeed, this may well explain why we found such low contact rates in our enclosure experiment—in order to carry a collar, individuals had to weigh > 40 g, which resulted in only adults being used; yet previous work has clearly demonstrated age‐related differences in movement and behaviour in adults compared to subadult individuals (Vanden Broecke et al. 2017; Mariën et al. 2020). While this work adds to growing evidence that simply assuming constant contact rates is inaccurate (Albery et al. 2025), additional, detailed research is necessary to understand how contact behaviour correlates with movement measures such as home range overlap based on both intrinsic and extrinsic drivers (Vander Wal et al. 2014).

This study suggests that both spatial and social factors are important for determining disease transmission in *M. natalensis* , with evidence of persistent spatial clustering and non‐equal distribution of contact rates between individuals. This adds further evidence that assuming homogenous mixing in transmission models would not accurately capture the transmission dynamics of MORV in *M. natalensis* (Borremans, Reijniers, Hughes, et al. 2017). Non‐linear transmission functions outperform linear transmission functions in most studies conducted over a range of natural host densities (Hopkins et al. 2020; Albery et al. 2025). Although Hopkins et al. (2020) found relatively few published examples, those that did exist were from a diverse range of systems suggesting that nonlinear transmission dynamics may be widespread. A more recent synthesis (Albery et al. 2025) provides clear evidence that saturating relationships between density and contact rates are common across diverse taxa, reinforcing the idea that non‐linear transmission dynamics are likely widespread. Misparametrising transmission functions can significantly bias estimates of density‐dependent pathogen transmission, particularly at high densities (Hopkins et al. 2020; Albery et al. 2025). As saturating contact–density relationships are common across systems (Albery et al. 2025), our findings support the idea that animals exhibit avoidance at high densities. This questions the reliability of home range overlap as a proxy for contact rates under such conditions and highlights the value of high‐resolution tools, such as miniature proximity loggers, for capturing fine‐scale behavioural data.

## Author Contributions

**L. Kirkpatrick:** conceptualization (lead), data curation (equal), formal analysis (lead), funding acquisition (equal), investigation (lead), methodology (lead), project administration (supporting), visualization (lead), writing – original draft (lead), writing – review and editing (lead). **J. Mariën:** conceptualization (supporting), data curation (equal), writing – review and editing (supporting). **C. Sabuni:** data curation (supporting), methodology (supporting), resources (supporting), writing – review and editing (supporting). **B. Mwamundela:** data curation (supporting), methodology (supporting), writing – review and editing (supporting). **H. Leirs:** conceptualization (supporting), data curation (lead), funding acquisition (lead), methodology (supporting), project administration (lead), resources (lead), writing – review and editing (supporting).

## Conflicts of Interest

The proximity loggers used in this study have since been developed into a spin‐off company by L.K. and H.L. (iosatracking.com).

## Supporting information


**Appendix S1:** ece372367‐sup‐0001‐AppendixS1.csv.

**Appendix S2:** ece372367‐sup‐0002‐AppendixS2.R.

**Appendix S3:** ece372367‐sup‐0003‐AppendixS3.docx.

## Data Availability

The full long term capture mark recapture dataset used in this study is available here: https://doi.org/10.1038/s41597‐023‐02700‐3. A subset of the data for the social–spatial models, which includes seroprevalence data and the code to rerun the analyses is included in the Supporting Information.
