# Supplementary material for: Partitioning Social and Spatial Drivers of Infection Risk
Source: Ecol Evol. 2025 Nov 2;15(11):e72367. doi: 10.1002/ece3.72367 (PMC12580238; doi:10.1002/ece3.72367)
Supplement: Supplementary file 3 — Appendix S3: ece372367‐sup‐0003‐AppendixS3.docx. [file ECE3-15-e72367-s002.docx]

Supplementary information:

Table 1: Full model constructions. A full model was fitted and refined to keep only those fixed terms that were deemed significant (if credible intervals around the estimate did not cross zero). The fixed terms were then tested with the inclusion of temporal (year and season) and spatial (both with and without temporal variation) effects. In all models, individual ID was fitted as a random term to account for repeated sampling of the same individual.

| Model name | Fixed parameters | Temporal parameters | Spatial parameters | Spatiotemporal parameters | Notes |
| --- | --- | --- | --- | --- | --- |
| Fixed full model | Reach, Betweenness, Degree, Vector, MinL, StrengthPC, Age, Observations, Density |  |  |  |  |
| Fixed reduced model | Degree, Strength, Age, Observations, Density |  |  |  |  |
| Fixed + Year | Degree, StrengthPC, Age, Observations, Density | Year (AR1) |  |  |  |
| Fixed + Season | Degree, StrengthPC, Age, Observations, Density | Season (seasonal) |  |  |  |
| Fixed + Spatial | Degree, StrengthPC, Age, Observations, Density |  | Spatial field |  |  |
| Fixed + Spatial + Seasonal | Degree, StrengthPC, Age, Observations, Density | Seasonal | Spatial field |  |  |
| Fixed + Spatiotemporal (IID) | Degree, StrengthPC, Age, Observations, Density |  |  | Spatiotemporal (IID) |  |
| Fixed + Spatiotemporal (EXC) | Degree, StrengthPC, Age, Observations, Density |  |  | Spatiotemporal (Exchangeable) |  |
| Fixed + Spatiotemporal (RW) | Degree, StrengthPC, Age, Observations, Density |  |  | Spatiotemporal (Random walk) | Excluded from Figure 2A as WAIC was very high |
| Fixed + Spatiotemporal (AR1) | Degree, StrengthPC, Age, Observations, Density |  |  | Spatiotemporal (AR1) |  |
| Spatial + Seasonal |  | Seasonal | Spatial field |  | Excluded from Figure 2A as WAIC was very high |
| Spatial |  |  | Spatial field |  | Excluded from Figure 2A as WAIC was very high |
| Seasonal |  | Seasonal |  |  | Excluded from Figure 2A as WAIC was very high |


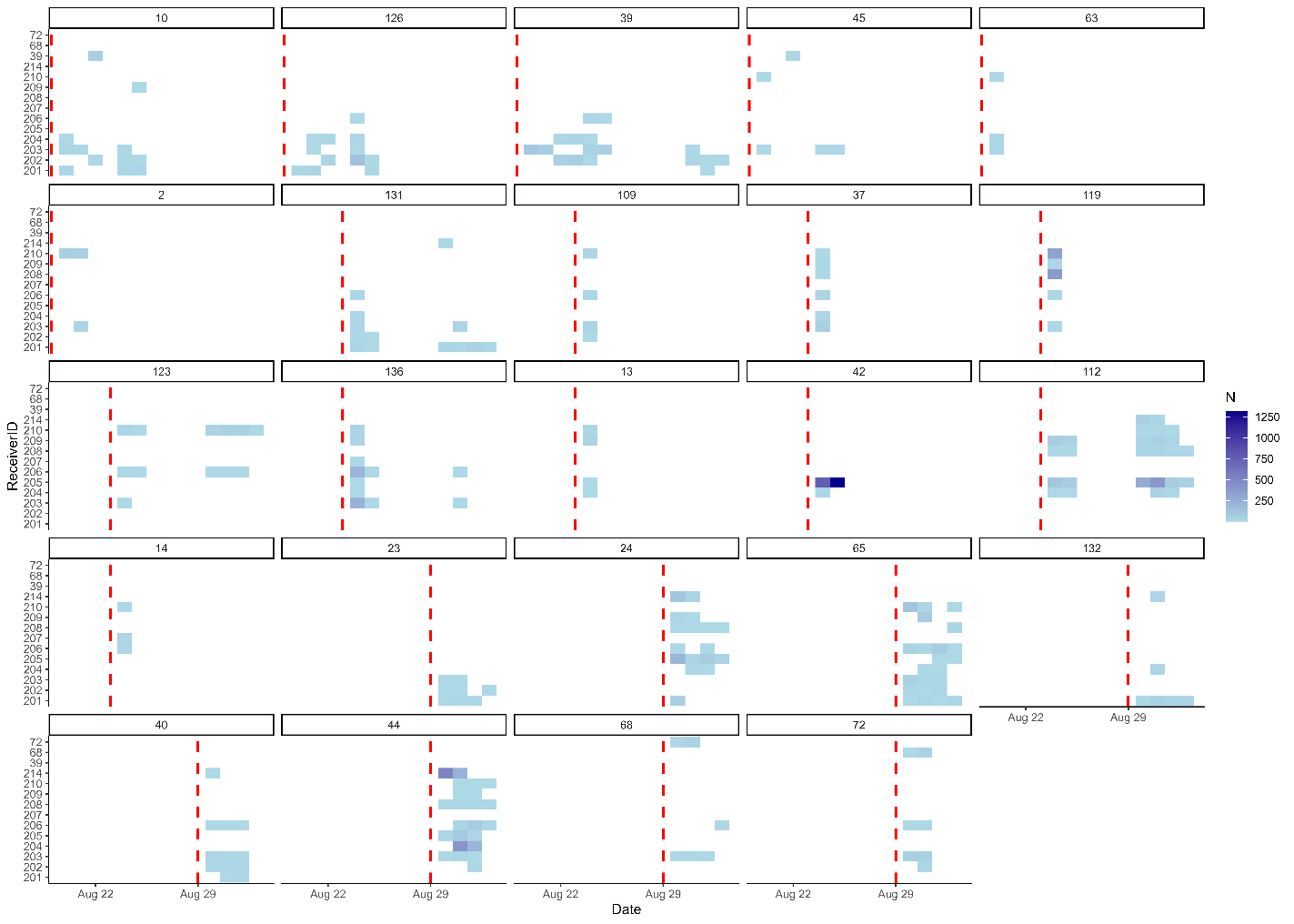


Figure 1: Logging distributions across the mobile and stationary loggers for the sampling periods. Y axis gives the receiver ID, X axis is the date, while the data is faceted by the ID of the mobile loggers. The darkness of the shading indicates the number of recorded logs. All loggers between 200 and 214 are stationary loggers, IDs below 200 are mobile loggers mounted on the rodents. The red dotted line indicates the release date. The mobile loggers are ordered by release date, with the first 6 being released first, followed by two separate batches of 12. We considered loggers that have only a day of logging as “failed”, and there were an additional 6 loggers that were never detected post release.

In our case, loggers failing does not necessarily mean a logger malfunction. Despite being covered with nets to prevent owl predation, our enclosures are open to other predators (cats, genets and snakes are the most prolific, with both cat and genet presence confirmed through camera trapping) and 4 loggers were recovered alongside evidence of a predation event (remains of the body). In these cases, the loggers were recovered by the daily sweeps of the arena, where the mobile phone app was used to scan for loggers. Other carcasses could have been removed completely by the predators. There is also the possibility that some rodents escaped the enclosures although every effort was made to prevent this. In addition, our stationary loggers did not fully cover the enclosure. The version of stationary loggers used in this experiment could not detect the mice underground, or when they were in the dense vegetation; therefore, there were gaps in coverage. This was confirmed by setting camera traps within the enclosure at points where we detected the mobile loggers with the mobile phone app but were out of range of the stationary loggers. In addition we see this in loggers where we had nights with no detections, between nights were loggers were detected, indicating that the mobile loggers were functioning but out of range of the stationary loggers.

The stationary loggers clearly demonstrate that some rodents moved across the grid during the nights, with detections at multiple poles, while others moved over a smaller area of the grid. Given the overlap in detections of mobile loggers at stationary loggers, which tells us that the mobile loggers are functioning, we do not see contacts between these mobile loggers. This suggests that while rodents are moving within the same area, they are avoiding coming into contact with each other, possibly through the use of auditory or olfactory cues.

Social contact logs were recorded between 5 of 30 mice and did not increase with density. The highest contact rates were during the low density period, and lowest were during the medium density period.


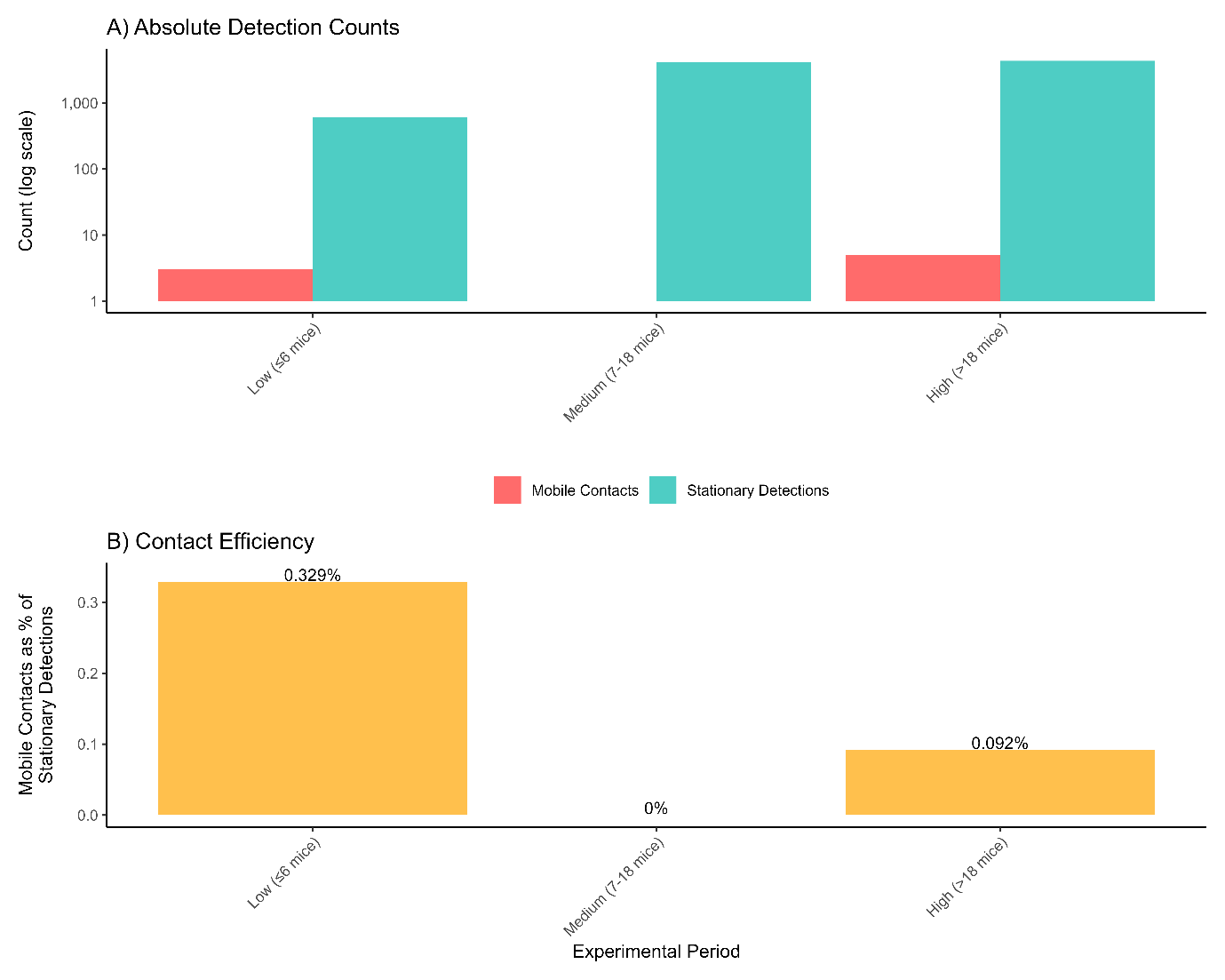


Figure S2 Caption: A) The distribution of detected contacts (logged) between mobile loggers (red bar) and mobile and stationary loggers (blue bar) for low, medium and high density groups. B) Contact efficiency for the different density groups comparing detections between mobile loggers as a percentage of detections by stationary loggers.
